# Sustained response of carcinoma ex pleomorphic adenoma treated with trastuzumab and capecitabine

**DOI:** 10.1186/1758-3284-2-12

**Published:** 2010-05-26

**Authors:** Elad Sharon, Ronan J Kelly, Eva Szabo

**Affiliations:** 1 Medical Oncology Branch, Center for Cancer Research, National Cancer Institute, National Institutes of Health, Bethesda, USA; 2 Lung and Upper Aerodigestive Cancer Research Group, Division of Cancer Prevention, National Cancer Institute, National Institutes of Health, Bethesda, USA

## Abstract

**Background:**

Carcinoma ex pleomorphic adenoma is a rare histologic subtype of salivary gland cancer with an overall poor prognosis. Limited histopathologic analyses have shown that some such tumors exhibit significant HER2/neu immunoreactivity, suggesting a potential role for HER2-based therapy. We report here a case of a 58-year old man with metastatic carcinoma ex pleomorphic adenoma who achieved a sustained long term response to combination therapy with trastuzumab and capecitabine.

**Case presentation:**

A 58 year old man presented with T1N2bM0 carcinoma ex pleomorphic adenoma and underwent surgery followed by adjuvant radiation therapy. Multiple metastases to bone were documented one year later. Since the original tumor was strongly HER2/neu positive by immunohistochemistry, the patient was treated with trastuzumab, capecitabine, and zoledronic acid. He experienced total resolution of symptoms and repeat FDG-PET scan after three cycles revealed interval disease resolution. Continued treatment has resulted in maintenance of disease control for over 2 years.

**Conclusion:**

This case illustrates the successful long term treatment of carcinoma ex pleomorphic adenoma with targeted therapy with trastuzumab in combination with chemotherapy. In the absence of definitive clinical trials which are unlikely to be performed due to the rarity of this tumor, case reports such as this one suggest potential utility for trastuzumab in combination with chemotherapy in the treatment of HER2/neu-overexpressing carcinoma ex pleomorphic adenoma.

## Background

Malignant salivary tumors represent 3 to 5% of all malignant head and neck tumors, with 2500 new cases diagnosed in the Unites States yearly [1]. Carcinoma ex pleomorphic adenoma is a rare, highly malignant tumor, accounting for 11.7% of salivary malignancies [2]. It usually develops from malignant transformation of a long-standing pleomorphic adenoma. Five-year survival for all stages of carcinoma ex pleomorphic adeonoma ranges from 30-76%, decreasing markedly for stage IV metastatic disease [2,3]. Only 27% of patients are alive one year after diagnosis of recurrence or metastasis.

There is no current chemotherapeutic standard for treatment of metastatic salivary carcinomas. Due to the infrequency of these tumors, the literature consists primarily of case reports and small phase II studies which combine multiple histologic subtypes. Objective response rates in the 14-30% range and rarely as high as 50% for cisplatin-based combinations have been reported [4]. Carcinoma ex pleomorphic adenoma has not been studied as a separate entity, and insufficient data exist regarding response to standard chemotherapy. This case shows a sustained response in a patient who was treated with trastuzumab and capecitabine.

## Case Presentation

**Figure 1:**
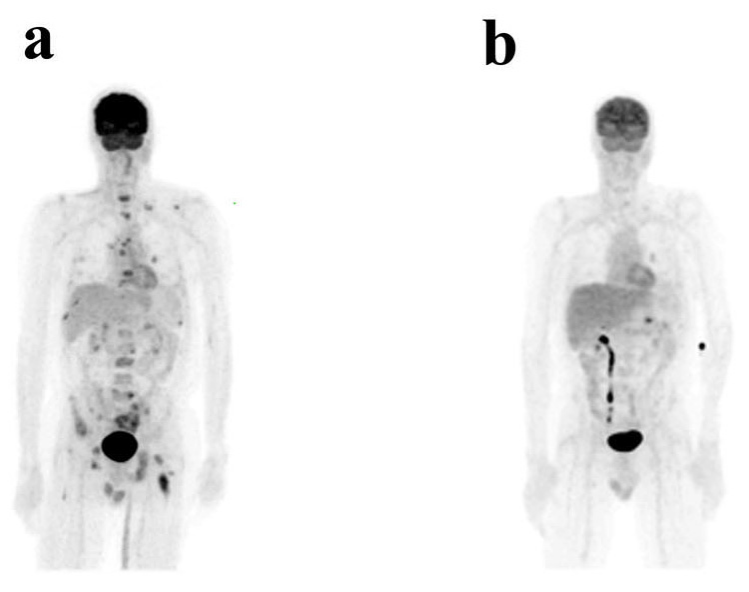
**Baseline (a) and 3 month post-treatment (b) FDG-PET scans**.

A 58 year-old man with a 44 pack-year history of smoking initially presented with a 3 year history of a painless right neck mass and new temperomandibular joint pain. Follow-up two months later after treatment with muscle relaxants revealed persistent pain and a new right facial nerve palsy. Physical exam revealed a 2 cm nontender palpable mass proximal to the right temperomandibular joint, while CT and FDG-PET also showed three enlarged hypermetabolic lymph nodes. Fine needle aspiration revealed poorly differentiated carcinoma. The patient underwent right total parotidectomy with sacrifice of cranial nerve VII. Tumor size was 2 cm, 4/18 lymph nodes (levels 2, 4, and 5) were involved by tumor, and the final pathologic diagnosis was carcinoma ex pleomorphic adenoma, high grade. The final stage was T1N2bM0. He received 60 Gy of radiation to the right parotid bed and involved lymph nodes. One year later, FDG-PET revealed multiple areas of increased bony uptake and bone biopsy confirmed disease recurrence (Figure 1a).

At the time of documented recurrence, immunohistochemical analysis of the original parotid resection revealed 3+ HER2/neu staining. Subsequent FISH analysis (performed after the patient was already on therapy) showed that the HER2/neu gene was not amplified. Given the strong immunostaining for HER2/neu and the known benefit of combining HER2-directed therapy with chemotherapy in HER2-overexpressing breast carcinoma, therapy was initiated with trastuzumab 6 mg/kg every 21 days and capecitabine 2000 mg/m^2 ^days 1-14 of a 21 day cycle [5]. Zoledronic acid 4 mg every 21 days was also initiated in view of the extensive bone involvement. The patient subsequently noted disappearance of bone pain and return to his baseline energy level. Repeat FDG-PET scan after three cycles revealed interval disease resolution with only physiologic uptake reported (Figure 1b). With continued treatment, clinical and radiological responses were sustained for greater than two years and were ongoing at the time of the last contact with the patient.

## Conclusions

Our case demonstrates the potential utility of using targeted therapy in combination with standard chemotherapy in HER2/neu-overexpressing carcinoma ex pleomorphic adenoma. There is a lack of knowledge regarding the role of targeted therapies in the treatment of salivary gland cancers. Recent data indicate that HER2/neu immunohistochemical expression is relatively common in carcinoma ex pleomorphic adenoma, occurring in 30-46% of cases [6,7]. However, clinical trials of HER2/neu targeted therapies in salivary carcinomas have been disappointing. Haddad et al. reported one extended response with trastuzumab in 14 patients with metastatic salivary gland cancers with HER2/neu expression, none of whom had carcinoma ex pleomorphic adenoma [8]. Similarly, a phase II trial of lapatinib in 62 patients with multiple histologic salivary carcinoma types with EGFR and HER2/neu expression also did not show any true responses [9].

However, two recent case reports, in addition to our patient, suggest that HER2/neu is a potential molecular target for a subgroup of patients with salivary gland tumors and strong HER2/neu immunoreactivity. Nashed et al. recently reported a durable complete response in a patient with ductal carcinoma arising in a pleomorphic adenoma who was treated with docetaxel and trastuzumab after initial stabilization with trastuzumab alone [10]. This patient's tumor strongly expressed HER2/neu, although FISH results were not reported. After initial disease stabilization with trastuzumab alone for five months, tumor progression was noted. Addition of docetaxel led to complete response of metastatic disease. A similar case report by Prat et al. confirmed this approach in a patient with metastatic salivary duct carcinoma who was successfully treated with trastuzumab in combination with carboplatin and taxol [11]. This patient's tumor, in contrast to our patient and the case described by Nashed and colleagues, apparently did not arise within a pleomorphic adenoma, but strongly expressed HER2/neu and was FISH-positive. Of interest, our patient's tumor was not FISH-positive despite being highly immunoreactive for HER2/neu. Nevertheless, all three cases of highly expressing HER2/neu salivary cancers show durable responses when trastuzumab was combined with any of three different chemotherapy regimens, suggesting that it may not matter which cytotoxic agent is added to trastuzumab to achieve a response. Since carcinoma ex pleomorphic adenoma is a very rare tumor and HER2/neu expressing salivary glad cancers of this (or another) subtype are relatively uncommon, it is unlikely that definitive clinical trials will be conducted. In the absence of such data, the case reported here, taken together with the two other above described cases, suggests that HER2/neu expression should be assessed in these salivary carcinomas and HER2-targeted therapies may provide significant clinical benefit in the appropriately selected patient subgroup.

## Consent

The patient has moved to another location and was unable to be contacted for consent.

## Competing interests

The authors declare that they have no competing interests.

## Authors' contributions

ESh provided the clinical case and has participated in the conception, design, and writing of the manuscript. RJK has participated in the manuscript writing. ESz provided the clinical case and has participated in the conception, design, and writing of the manuscript.

All authors approved the final manuscript.
